# Disruption of estrogen signaling by developmental exposure to BPA and TBT causes long term functional deficits in zebrafish retina

**DOI:** 10.3389/fphar.2026.1750796

**Published:** 2026-02-17

**Authors:** J. S. Jensen, L. Ouellette, R. Harris, P. Owrang, V. P. Connaughton

**Affiliations:** 1 Department of Biology, American University, Washington, DC, United States; 2 Department of Neuroscience, American University, Washington, DC, United States

**Keywords:** bisphenol A, *Danio rerio*, ERG, estrogen, tributyltin

## Abstract

Bisphenol A (BPA) and tributyltin (TBT) are two endocrine disrupting compounds (EDC) that have opposite effects on estrogen signaling. BPA is an estrogen agonist that binds to all estrogen receptor types. TBT is an aromatase inhibitor that binds to the enzyme aromatase, preventing the synthesis of estrogen from testosterone. Both estrogen receptors and aromatase are localized to the retina and estrogen signaling is required for proper eye and retinal neurogenesis. Abnormal eye growth and retinal changes are reported immediately after developmental exposure to either EDC consistent with the role of estrogen in proper neurogenesis. In this review, we examine the impact of BPA and TBT exposure on the development and function of the visual system. We focus primarily on zebrafish but include data from other species to show trends across vertebrates. We discuss a case study designed to determine if a transient developmental exposure to BPA or TBT has persistent effects that are evident in adults and if these latent outcomes reflect the opposite impact of these compounds on estrogen signaling. Surprisingly, although some opposing outcomes were observed, most differences in adult retinal function were similar between the two compounds, with varying effects noted by concentration and exposure age. Overall, we conclude that developing zebrafish retina is sensitive to EDCs that target estrogenic pathways. However, these findings cannot be explained by estrogenic modulation alone, suggesting additional mechanisms beyond their current established roles.

## 1 Introduction

Endocrine disrupting compounds (EDCs) are natural or synthetic compounds (Niehs, 2025) structurally similar to natural hormones (Ahn and Jeung, 2023). EDCs can influence the synthesis, action, and/or downstream signaling pathways of hormones (Bertram et al., 2022; Ahn and Jeung, 2023) by either blocking enzyme activity, antagonizing receptors, or mimicking endogenous ligands (Wingfield and Mukai, 2009; De Coster and van Larebeke, 2012). Exposure is a significant public health concern as EDCs are linked to certain cancers and cardiovascular problems, as well as metabolic, reproductive, and neurological disorders (Kumar et al., 2020; Bertram et al., 2022; Ahn and Jeung, 2023). Early life/developmental stages are the most sensitive to EDC exposure, with both immediate (Kahn et al., 2020; Puri et al., 2023) and latent/later life effects reported (Kumar et al., 2020; Duh-Leong et al., 2023). Research has shown that neurodevelopment is particularly vulnerable to EDC exposure (Damiano et al., 2025).

EDCs are prevalent and persistent contaminants in aquatic environments (Le Page et al., 2011; Soffker and Tyler, 2012) with landfills, wastewater effluent, and agricultural/road/roof runoff serving as primary sources (Kumar et al., 2020; Bertram et al., 2022). Human exposure occurs significantly via ingestion and inhalation; fetal exposure also occurs as many EDCs can cross the placenta (Bertram et al., 2022; Ahn and Jeung, 2023). Non-human animal exposure occurs through ingestion, inhalation, and/or uptake across the gills (Bertram et al., 2022). Importantly, low doses of EDCs are reported to have significant effects on both humans and animal models (Puri et al., 2023), suggesting non-linear and non-monotonic effects of these compounds (Kumar et al., 2020; Duh-Leong et al., 2023).

Many of the identified EDCs target estrogen signaling pathways, resulting in significant effects on both development and adult reproductive physiology (Mericskay et al., 2005; Dickerson et al., 2011; Jasarevic et al., 2011; Meeker, 2012; Damiano et al., 2025). Some EDCs specifically interfere with estrogen synthesis; while others bind estrogen receptors, resulting in aberrant downstream signaling and gene transcription. Outside the reproductive axis, proper estrogen signaling is important for neurogenesis (Wesselman and Wingert, 2024) and EDCs affecting estrogen pathways have both overt and subtle effects on the visual system (Dong et al., 2006; Hamad et al., 2007; Wang et al., 2012).

In this review, we focus on the impact of EDC exposure on development and function of the visual system. We discuss two well-known compounds–bisphenol A and tributyltin–which have opposite effects on estrogen signaling. Bisphenol A (BPA) is a weak estrogen agonist (Chapin et al., 2008) that increases estrogen-dependent signaling. Tributyltin (TBT), on the other hand, prevents the synthesis of estrogen (estradiol or E_2_) from testosterone (Matthiessen and Gibbs, 1998; McAllister and Kime, 2003; McGinnis and Crivello, 2011). Given their contrasting effects, we examine if developmental exposure to environmentally relevant levels of BPA and TBT cause opposing functional differences in the visual system. We have structured our review to provide background on both BPA and TBT, discuss estrogen signaling within retina, summarize reported effects of EDC exposure on retinal development and function, and then review our recent findings as a case study. Though our work focuses on zebrafish (*Danio rerio*), we also include data from other species to show trends across vertebrates.

## 2 Bisphenol A (BPA)

BPA is a synthetic compound widely used in the manufacturing of industrial/household plastics, epoxy resin, and the lining of food and drink containers (Geens et al., 2012). As an environmental pollutant, BPA leaches into aquatic environments from industrial and material waste, leading to long term consequences to the local environment’s food web. On top of the environmental impact, human exposure to BPA is common (Rubin, 2011). There are several recent, comprehensive reviews of BPA’s negative effects on human health (Lin et al., 2023; Hyun and Ka, 2024; Triswindyaningrum et al., 2025).

Human exposure to BPA can occur via inhalation, ingestion, or dermal contact (Lin et al., 2023; Hyun and Ka, 2024), though the primary pathway is through ingestion, as BPA leaches from food containers, canned goods, and thermal papers into food products (Geens et al., 2012; Hyun and Ka, 2024). Human exposure is significant, with over 90% of people worldwide showing measurable levels of BPA in their tissues (Lin et al., 2023; Triswindyaningrum et al., 2025) and recent estimates suggest the world average daily human intake of BPA is 2.53 µg/p/day (Wang et al., 2020). BPA is readily absorbed in the gastrointestinal tract due to its lipophilicity and undergoes metabolism in the liver, forming BPA-glucuronide and BPA-sulfate (Almeida et al., 2018). Although these conjugates are traditionally considered inactive, deconjugation can occur via tissue-specific enzymes or gut microbiota, regenerating biologically active BPA (Dekant and Volkel, 2008). BPA and its metabolites can cross the placenta (Almeida et al., 2018), with detectable levels in the fetal circulation, amniotic fluid, breast milk, and neonatal urine, highlighting particular risk during early development (Vandenberg et al., 2007). Humans exposed to BPA before birth show increased anxiety and depression as children (Hyun and Ka, 2024) and *in utero* exposure, particularly during the third trimester, is associated with an increased risk of preterm births (Lin et al., 2023).

A central concern regarding BPA toxicity is the substantial evidence that low concentrations can induce significant biological effects (Vandenberg et al., 2012) and the life stages that are most sensitive are embryonic and early postnatal periods (Rubin, 2011). Low-dose BPA exposure has been linked to altered neurobehavior, metabolic disturbances, reproductive tract abnormalities, and long-term physiological deficits across numerous vertebrate models (Rubin, 2011; Pironti et al., 2021; Hyun and Ka, 2024) and in humans (Mustieles et al., 2015; Lin et al., 2023; Hyun and Ka, 2024). These outcomes identify a non-monotonic dose response relationship for BPA (Vandenberg et al., 2012; Lagarde et al., 2015). Importantly, environmentally relevant BPA exposures often fall within the low-dose range where non-monotonic responses are most prominent, increasing ecological and human-health relevance.

Although BPA is not as persistent as legacy contaminants like polychlorinated bisphenols (PCB), another EDC, continuous release from wastewater, plastic degradation, and landfill leachate has resulted in widespread and persistent environmental contamination (Almeida et al., 2018). BPA concentrations in streams range from 0.14 to 12 μg/L. BPA levels measured in human urine samples range widely (0.4–149 μg/L) with a geometric mean of 2.6 μg/L (Vandenberg et al., 2010). BPA accumulates in aquatic systems, where it affects both micro- and macro-organisms by altering growth, reproduction, and endocrine function (Pironti et al., 2021). In fish and amphibians, for example, chronic BPA exposure has been associated with disrupted gonadal development, abnormal steroidogenesis, impaired reproductive behavior, and long-term physiological deficits (Corrales et al., 2015). These findings suggest that BPA poses significant risk not only to individual organisms but also to broader ecosystem structure and function.

## 3 Tributyltin (TBT)

TBT has industrial uses in antifouling paints, lumber and textile production, and as a stabilizer in PVC production. Its use on marine structures and ship hulls allows it to readily leach into the water, where it quickly settles and adheres to marine sediment, creating a bioavailable sink (Hoch, 2001). This sequestration prevents degradation and increases the risk of chemical resuspension, especially around major ports where there is increased ship traffic. In 2008, the International Convention of the Control of Harmful Antifouling Systems on Ships (AFS Convention–International Maritime Organization) banned the use of TBT on registered ships worldwide. Despite global restrictions, regulations are not strictly enforced, and TBT based paints are still being produced and are available for purchase (Uc-Peraza et al., 2022). Measurable TBT levels have been found along the coasts of several different countries. Major ports are especially susceptible, but alarmingly, some environmentally protected areas are just as contaminated (Castro et al., 2021). Researchers found that 77% of samples collected from marine protected areas along the Latin American coast had TBT levels ranging from 0.002–0.05 μg/g (Castro et al., 2021). TBT levels collected from the surface waters of India and China, reached 0.342 and 0.977 μg/L respectively (Gui-bin et al., 2001; Garg et al., 2011). In Brazil, sediment samples collected from Vitoria range from 0.006–0.0211 µg Sn/g dry weight (Abreu et al., 2021) and in Ceará, samples collected after dredging activities reached 0.0526 µg Sn/g dry weight (Moreira et al., 2021).

Out of all organotin compounds, TBT is regarded as the most toxic due to its slow degradation, environmental persistence, bioaccumulation potential, and ability to cross the blood brain barrier (Rouleau et al., 2003; Al-shatri et al., 2015). Human exposure to TBT occurs through dietary intake, direct exposure to treated products, and dust inhalation (Sousa et al., 2014). Cohort studies examining human placentas obtained after birth have detected measurable levels of TBT in Scandinavian countries (Rantakokko et al., 2013; Rantakokko et al., 2014). A Danish study specifically discovered that the human placental concentration of TBT was inversely associated with thyroid hormones (Li et al., 2018), which are critical for fetal development. Exposure to TBT during gestation in humans has been linked to reduced fetal weight and placental abnormalities (Adeeko et al., 2003; Liu et al., 2021). And more recently, the impact of TBT on mammalian cells was reviewed by (Correia et al., 2025).

## 4 Estrogen receptor types and signaling mechanism

Estrogen is present in three forms in vertebrates: E_1_ (estrone), E_2_ (estradiol; 17β-estradiol), and E_3_ (estriol) (Bondesson et al., 2015; Nazari and Suja, 2016). E_2_ is the major biological form in reproductively active females (Nazari and Suja, 2016). All estrogens are synthesized from cholesterol, with the final step in E_2_ synthesis being the aromatization of testosterone by the enzyme aromatase (Simpson et al., 2002; Nuzzi and Caselgrandi, 2022). Circulating E_2_ binds to one of several intracellular estrogen receptors (ER), which are ligand-activated transcription factors. Activated (ligand-bound) ERs then dimerize and bind to estrogen response elements (ERE) on DNA, affecting gene transcription and/or rapidly altering membrane potentials (Belcher and Zsarnovszky, 2001; Cascio et al., 2015). In mammals and birds, there is a single aromatase gene (*cyp19a1*) (Bondesson et al., 2015; Cascio et al., 2015); whereas, in zebrafish there are two aromatase genes, *cyp19a1a* (*aromatase A*, gonadal aromatase) and *cyp19a1b* (*aromatase B*, brain aromatase) (Chiang et al., 2001; Wesselman and Wingert, 2024). Estrogen binds to two ER isoforms in mammals: ERα and ERβ (Rubin, 2011; Rochester, 2013). In zebrafish, there are three ER isoforms, one for ERα (*esr1*) and two for ERβ (ERβ1 or *esr2b* and ERβ2 or *esr2a*) (Menuet et al., 2002; Menuet et al., 2004; Bondesson et al., 2015) and zebrafish ERs have high sequence homology with mammalian ER (Lassiter et al., 2002; Wesselman and Wingert, 2024). E_2_ can also bind to a membrane-bound G-protein coupled estrogen receptor (GPER), triggering different intracellular cascades (Belcher and Zsarnovszky, 2001) involving MAPK/ERK and P13K/AKT signaling pathways (Lim et al., 2002; Gorelick et al., 2008; Bouskine et al., 2009; Cohen et al., 2022). All vertebrates have a single copy/isoform of GPER (Bondesson et al., 2015).

Most of the circulating E_2_ is synthesized in the ovaries (Nazari and Suja, 2016). However, local synthesis can also occur. The aromatase enzyme is present in brains of mammals, birds, fish, and amphibians (Pelligrini et al., 2005; Bondesson et al., 2015). In mammals and birds aromatase is localized to nerve cells; in fish, particularly zebrafish, aromatase is localized to radial glial cells in the brain (Menuet et al., 2005). Aromatase-positive radial glial cells are progenitor cells (Chapouton et al., 2010) that proliferate throughout life in the fish and developmental aromatase expression is estrogen-dependent (Kishida et al., 2001; Menuet et al., 2005; Sawyer et al., 2006; Vosges et al., 2010; Chung et al., 2011; Brion et al., 2012).

### 4.1 BPA and TBT have opposite effects on estrogenic mechanisms

BPA binds to both ERα and ERβ (Rubin, 2011; Rochester, 2013), mimicking endogenous estrogen, though BPA is reported to have greater affinity for ERβ (Ben-Jonathan and Steinmetz, 1998; Vandenberg et al., 2009). Overall, for both ERs, the affinity for BPA is weak compared to E_2_, though BPA binding can induce transcription of estrogen responsive genes (Kishida et al., 2001; Richter et al., 2007; Chung et al., 2011; Kinch et al., 2015; Cano-Nicolau et al., 2016; Qiu et al., 2016). Beyond this classic genomic pathway, BPA can also bind GPER (Mustieles et al., 2015), initiating rapid non-genomic responses (Lim et al., 2002; Gorelick et al., 2008; Bouskine et al., 2009). The ability of BPA to interact with estrogen receptors is suggested to be causal for some adverse effects in humans (Almeida et al., 2018).

The aromatase enzyme binds testosterone and catalyzes the formation of E_2_ (Simpson et al., 2002; Nuzzi and Caselgrandi, 2022). Aromatase is also able to synthesize E_1_ from androstenedione, with the resulting E_1_ being converted to E_2_ (Luu-The, 2013). Thus, EDCs that bind aromatase disrupt E_2_ synthesis. TBT is one of these compounds. As an aromatase inhibitor, TBT reversibly binds to the active site on aromatase, preventing substrate (testosterone) binding (Cheng and Yang, 2023). Consequently, E_2_ levels are reduced and testosterone levels are increased after TBT exposure (McGinnis and Crivello, 2011) which causes masculinization in gastropods (Horiguchi, 2006) and zebrafish (McAllister and Kime, 2003; Santos et al., 2006; McGinnis and Crivello, 2011). TBT concentrations as low as 0.003–0.005 μg/L have deleterious consequences for marine and freshwater organisms (Al-shatri et al., 2015). Filter and sediment feeding organisms are especially at risk and TBT has been detected in both target and non-target organisms (Hoch, 2001).

### 4.2 Estrogen in retina

The general structure and function of the vertebrate retina is highly conserved (Baden et al., 2020). Rod and cone photoreceptors have their cell bodies in the distal outer nuclear layer (ONL) and synapse onto second-order bipolar and horizontal cells in the outer plexiform layer (OPL). Bipolar cell somata are in the inner nuclear layer (INL) and these cells are presynaptic to amacrine and ganglion cells in the inner plexiform layer (IPL). Ganglion cell axons leave the eye, forming the optic nerve. Signaling from photoreceptors to bipolar cells to ganglion cells is direct and glutamatergic; horizontal and amacrine cells, with cell bodies in the INL, are local circuit neurons that provide inhibitory feedback to the photoreceptor-bipolar-ganglion cell pathway in the OPL and IPL, respectively. Primate retinas, including humans, have three cone types (L, Long wavelength sensitive or “red” cones | M, Mid-wavelength sensitive “green” cones | S, Short wavelength sensitive or “blue” cones) (Mustafi et al., 2009), non-primate mammals have two cone types (M and S) (Lindenau et al., 2019), and zebrafish have four cone types (L, M, S, and a UV sensitive cone) (Robinson et al., 1993). Multiple bipolar, horizontal, amacrine, and ganglion cell types are reported in all species, contributing to complex and diverse levels of processing within the retina (Baden et al., 2020).

While circulating E_2_ can reach the retina through the blood (Chaychi et al., 2015), it is also locally synthesized (Nuzzi et al., 2018). In rats, aromatase is found in distal retinal neurons (photoreceptors, horizontal and bipolar cells) within the ONL, OPL, and INL (Cascio et al., 2015; Valero-Ochando et al., 2024). Goldfish retina contains aromatase-positive horizontal, bipolar, and amacrine cells, as well as aromatase-containing ganglion cell axons (Gelinas and Callard, 1993; Callard et al., 1995). Aromatase transcripts have been identified in zebrafish (Sawyer et al., 2006), along with *esr1* and *esr2b* (Cohen et al., 2025). Inhibition of aromatase changes the thickness of the IPL, INL, and OPL in both zebrafish and rats (Salyer et al., 2001; Hamad et al., 2007).

E_2_ exposure affects photoreceptor gene expression (Hao et al., 2013; Cohen et al., 2022), suggesting ER are present in that retinal layer. ERα, ERβ, and GPER are present in mouse retina, with GPER (Jiang et al., 2019; Li and Li, 2020a; Li and Li, 2020b; Pinon-Teal and Ogilvie, 2024) and ERβ expressed on ganglion cells (Rodriguez-Ramirez et al., 2025). In rat and human retinas, ERα is expressed in the OPL and in amacrine and ganglion cells in inner retina and ERβ is expressed in the IPL, with colocalization of these ER types observed on some amacrine and ganglion cell types (Cascio et al., 2015). GPER has been identified in the retinas of zebrafish (Liu et al., 2009; Jayasinghe and Volz, 2012; Shi et al., 2013) and goldfish (Mangiamele et al., 2017).

While estrogen is present in both males and females, the higher circulating E_2_ levels in females are suggested to underlie sex-differences in retinal function. ERG recordings from female Sprague-Dawley rats (age 2–6.5 months) are larger in amplitude than age-matched males (Chaychi et al., 2015). Similarly, multifocal ERGs recorded from men and women <50 years of age identified shorter implicit times in women participants (Ozawa et al., 2014). Increased E_2_ levels in female Tungara frogs during the reproductive season is associated with an increase in retinal sensitivity (Leslie et al., 2021). Estrogen is also neuroprotective in retina (Bondesson et al., 2015; Cascio et al., 2015). E_2_ helps regulate the blood-retinal-barrier and protect photoreceptors from glutamate-induced damage (Nuzzi and Caselgrandi, 2022). In a mouse retinopathy of maturity model, activation of GPER on ganglion cells and astrocytes decreased endoplasmic reticulum stress response to hypoxia (Li and Li, 2020a) and decreased apoptosis (Li and Li, 2020b). Activation of GPER on mouse ganglion cells was also protective against NMDA-mediated neurotoxicity (Jiang et al., 2019); while activation of ERβ was protective after optic nerve crush (Rodriguez-Ramirez et al., 2025). *In vitro*, cultured mouse Muller glial cells were protected from oxidative stress by E_2_ treatment (Tawarayama et al., 2024). Given this latter role of E_2_, it is not surprising that age-related changes in estrogen levels are associated with neurodegenerative retinal diseases in humans (Gupta et al., 2005; Nuzzi et al., 2018) and rodents (Rowe et al., 2025) or that estrogen modulation as a breast cancer treatment has been associated with a variety of retinal complications (Eisner et al., 2008; Eisner and Luoh, 2011; Moschos et al., 2012).

## 5 EDC exposure and retinal development

Retinal development has been extensively studied in zebrafish because their rapid external development and transparent eggs allow a real-time observation of events which are difficult to do in mammals (Kimmel et al., 1995; Phillips and Westerfield, 2014). The initial work documenting photoreceptor development (Branchek and Bremiller, 1984), followed by electron microscopic analysis (Schmitt and Dowling, 1994; 1999), identified that zebrafish retinal development begins at 24 hpf (hours post fertilization). At 32 hpf ganglion cell axons project to the brain (Stuermer, 1988) and by 72 hpf, the larvae have hatched and all retinal layers are present (Schmitt and Dowling, 1999). One day later (96 hpf), optokinetic responses, a retina-based response where the eyes track a moving stimulus, can be recorded. By 7 dpf, zebrafish have exhausted their yolk sac, begin feeding, and visually guided optomotor responses can be recorded (Chiang et al., 2001; Neuhauss, 2003; Muto et al., 2005).

Development of estrogen signaling occurs in parallel with zebrafish retinal development. Zygotic synthesis of ER mRNA begins ∼24–48 hpf (Bardet et al., 2002; Lassiter et al., 2002; Tingaud-Sequeira et al., 2004; Mouriec et al., 2009a). *Cyp19a1b* is detected at 32 hpf in larvae with expression upregulated by E_2_ treatment at ∼25 hpf (Balshaw, 2023). All zebrafish ER types are expressed and functional in brain at 24–26 hpf (Balshaw, 2023). GPER mRNA can be identified in the eye ∼24–26 hpf (Jayasinghe and Volz, 2012; Shi et al., 2013). At 5 dpf, aromatase protein can be detected in ganglion cells and in the INL using immunocytochemistry (Ulhaq and Kishida, 2026).

There is a similar overlap of estrogen and eye development in mammals. In mice, ER expression begins at E (embryonic day) 9.5 when ERα is identified in the heart. ERβ is identified in the brain beginning at E10.5 and remains the only ER expressed in that tissue until E16.5 when ERα expression was identified (Lemmen et al., 1999). The presumptive neural retina is distinguished in the optic cup at ∼E9.5. From E11 to E18 neurogenesis of retinal ganglion cells, horizontal cells, amacrine cells, and cone photoreceptors occurs and the first ganglion cell axons leave the eye ∼ E11.5. A second wave of retinal neurogenesis occurs from postnatal day (P) 0 – P7 when rods, bipolar cells, and Muller glia are formed (Zhang et al., 2011; Heavner and Pevny, 2012). In humans, the presumptive neural retina forms around 4–5 weeks of gestation and retinal differentiation begins ∼47 days with rods and cones evident at weeks 10–15 (Graw, 2010). At 9 weeks, the placenta is the major source of fetal estrogen; fetal ERα and ERβ transcripts are identified at 13 weeks (Bondesson et al., 2015).

Given the overlap in timing, it is not surprising that developmental disruption of estrogen signaling either by blocking aromatase activity or antagonizing estrogen receptors has adverse effects on retinal/eye development. Exposure to aromatase inhibitors from 24 to 120 hpf thins ONL, IPL, and GCL in zebrafish (Hamad et al., 2007). Morpholino knockdown of *aromatase B* (*cyp19a1b*) decreased overall eye and optic nerve development, reduced thickness of the INL and the IPL, increased apoptosis in the eye, and caused deficits in visual based behaviors. These effects were mediated by ERβ (Ulhaq and Kishida, 2026). Aberrant activation of GPER causes concentration-dependent effects on survival and morphology during zebrafish embryogenesis (Jayasinghe and Volz, 2012). Larval exposure to 4-OH-A, an aromatase inhibitor, reduced the thickness of retinal layers (Hamad et al., 2007) and caused visual deficits in adults (Gould et al., 2017). In another teleost, medaka, adult exposure to EE_2_, a potent estrogenic EDC, caused generational effects that delayed eye pigmentation in larvae and changed expression of the genes associated with synaptic structure, synaptic transmission, and eye structure and development (Qin et al., 2023).

Polychlorinated biphenyls (PCBs) are another well-known EDC (Ulbrich and Stahlmann, 2004) that are estrogenic (Ma and Sassoon, 2006), able to bind ER (Damiano et al., 2025), and effect eye development. Examination of retina morphology and ultrastructure in zebrafish larvae exposed to PCB1254 from 0 to 96 hpf revealed delayed retinal layer development and smaller photoreceptor outer segments at 72 hpf and irregularly arranged photoreceptors and larger photoreceptor and ganglion cell layers at 96 hpf (Wang et al., 2011). Zebrafish larvae exposed to PCB1254 until 7 dpf displayed concentration-dependent decreases in optomotor responses and in the expression of photoreceptor-specific genes (Zhang et al., 2015). Adult female offspring of pregnant Long-Evans rats fed either PCB77 or PCB47 showed decreased scotopic ERG b-wave amplitudes at high illuminance levels (for PCB77) and increased b-wave latency at low illuminance levels (for PCB47) (Kremer et al., 1999). Immunocytochemical analysis of brains of adult offspring of pregnant Sprague Dawley rats fed a mix of 14 different PCB congeners during pregnancy and lactation revealed region-specific reductions in endothelial cell size, increases in GAD67 immunoreactivity, and decreased lipofuscin autofluorescence (Rustom and Reynolds, 2025).

### 5.1 Specific effects of BPA, TBT, and related compounds on retinal development

Developmental exposure to both BPA and TBT cause immediate effects on neurogenesis, with many of the studies performed in zebrafish. Early BPA exposure reduces zebrafish eye diameter (Crowley-Perry et al., 2021; Volz et al., 2024) and affects retinal layer thickness (Volz et al., 2024). Zebrafish larvae exposed to BPA showed changes to red (L) and UV cones within the retinal mosaic (Qiu et al., 2025). Chronic 8 days BPA exposure thinned the IPL and impaired responses to red and green (M cone) light (Volz et al., 2024). Similarly, adult male zebrafish exposed to BPA for 7 weeks showed altered color preference (Li et al., 2017). BPA increased ERα and ERβ expression, but decreased locomotor behavior in larvae (dos Santos et al., 2022). BPA analogs also alter visual function. Chronic BPS exposure until 6 dpf reduced thickness of the ganglion cell layer (Gu et al., 2019) and BPS exposure for 120 days disorganized the ONL, thinned the IPL and GCL, and altered adult spectral sensitivities (Liu et al., 2018). BPS exposure from 2 to 5 dpf disrupts spacing and alters signaling of red and UV cones (Qiu et al., 2023); differences in optic nerve structure were noted in a separate study that chronically exposed larvae until 6 dpf (Gu et al., 2019). Exposure to TBBPA until 5 dpf decreased both eye size and optokinetic responses in zebrafish (Baumann et al., 2016).

TBT exposure during early life stages causes abnormal and delayed eye development (Hano et al., 2007), thinning of the cornea (Wang and Huang, 1999), and retina-specific defects (Fent and Meier, 1992; Wang and Huang, 1999; Wester et al., 2004; Dong et al., 2006).Chronic 5 days TBT exposure (0–120 hpf) increased apoptosis in zebrafish retina, with the greatest difference observed at 60 hpf when there was a 2× increase in macrophages (Dong et al., 2006). In tropical guppies, a 7 days exposure to TBT decreased retinal pigment epithelium, disorganized the photoreceptor layer, and caused vacuoles in the IPL (de Paulo et al., 2020). In minnows, embryonic-larval exposure to TBT similarly reduced the amount of pigment and disrupted retinal layering (Fent and Meier, 1992) and a 7 days exposure decreased retinomotor responses in tiger perch (Wang and Huang, 1999). TBT exposure increased oxidative stress in the eyes of medaka juveniles (Shi et al., 2021) and disrupted the blood-brain-barrier in rats (Mitra et al., 2013; Mitra et al., 2015). Eyes of neonatal ICR mice exposed to trimethyltin (TMT), a related organotin compound, until P14 had thinner retinal layers, increased apoptosis, increased overall glutamate levels, and decreased micro-ERG b-wave amplitude, consistent with neurotoxicity (Kim et al., 2023). TMT exposure during zebrafish development caused concentration-dependent effects that reduced vision-based behaviors, thinned retinal layers, formed pyknotic nuclei, and distorted the boundaries of the IPL and OPL (Kim et al., 2019). A 14-day exposure to Triphenyltin (TPT) altered expression of genes involved in polarity during retinal development in zebrafish (Li and Li, 2020a).

## 6 Case study: Does transient early life BPA/TBT exposure cause persistent effects in zebrafish

### 6.1 Premise and experimental design

Most published reports of BPA and TBT induced effects typically occurred immediately after exposure, and exposure was often chronic, lasting multiple days. In contrast, our approach assessed whether a transient, 24 h developmental exposure to either compound was sufficient to cause long-term changes in adult retinal function and anatomy. We also sought to determine if these compounds have opposite sequelae in retina, given their contrasting effects on estrogen signaling.

Compound exposure occurred when larvae were either 3 dpf or 7 dpf because these ages represent key time points in both visual system and estrogen pathway development, as noted above. Exposure lasted for 24 h. The concentrations used for each compound are environmentally relevant and in agreement with previous studies (Fent and Meier, 1992; Kolpin et al., 2002; Schmidt et al., 2004; el Hassani et al., 2005; Schmidt et al., 2005; Dong et al., 2006; Oliver et al., 2011; Saili et al., 2012; Wang et al., 2013; Borges et al., 2014; Hayashi et al., 2015; Kinch et al., 2015; Weber et al., 2015; Cano-Nicolau et al., 2016; Santangeli et al., 2016). Our BPA concentrations were 0.228 μg/L (0.001 µM) and 22.8 μg/L (0.1 µM), with DMSO (0.0003%) as the vehicle control (Cohen et al., 2025); TBT concentrations were 0.04 μg/L (0.12 nM) and 0.4 μg/L (1.2 nM), with 0.1% ethanol as the vehicle control (Jensen et al., 2025). After the 24 h exposure, treated larvae were returned to system water and allowed to grow undisturbed until adulthood (≥3–4 months of age). In adults, we examined retinal anatomy (using H&E staining of retinal sections) and function [using electroretinograms (ERGs)] to identify if there were persistent outcomes resulting from transient developmental exposure.

### 6.2 Early life exposure to either BPA or TBT altered adult retinal function

To determine if developmental exposure to BPA or TBT altered retinal function, we used ERGs. The ERG response components include an initial negative a-wave (photoreceptor response) at light ON, followed by a positive b-wave (ON-bipolar cell response), and a positive d-wave (OFF-bipolar cell response) at the end of the light pulse (Perlman, 2007; Nelson and Singla, 2009). We decided to focus on ERGs as differences in b- and d-waves (reflecting ON and OFF bipolar cell responses) have been associated with retinal diseases in animal models (Constable et al., 2023; Aviles et al., 2025; Medina Arellano et al., 2025) and in the clinic (Cornish et al., 2021; Chiang et al., 2022; Constable et al., 2023).

ERGs were recorded from adult zebrafish that had been developmentally exposed to either BPA or TBT when they were 3 dpf or 7 dpf. ERGs were recorded as described in (Nelson and Singla, 2009; Cohen et al., 2025; Jensen et al., 2025). Briefly, eye cups, with the cornea and lens removed, were placed onto a piece of 0.45 µm black filter paper in the recording chamber and perfused (0.3 mL/min inflow; 4 mL/min outflow) with MEM solution that had been equilibrated with 95%O_2_/5%CO_2_. A tungsten recording electrode was placed into the eye cup to record responses to 300 ms flashes of white light presented individually at 7 different brightness levels (ND 3.0 – ND 6.0 in 0.5 ND increments) on an infrared background (RG780 filter). Each flash was presented 4 times for each brightness level, and the entire protocol was repeated 10 times for a total of 280 retinal responses per eye. Recordings were amplified using a DAM80 amplifier (WPI) and Digidata 1440A (Axon Instruments). Data was collected using pCLAMP software (Axon) and analyzed in Origin. For each recording, b-wave amplitudes were measured from the a-wave trough to the peak of the b-wave (50–200 ms after stimulus onset); d-wave amplitude was measured as the peak occurring within 25–300 ms after stimulus offset. Technical replicates were excluded from analysis if b-waves were negative or the response occurred outside the time intervals. Amplitudes and implicit times (= time to peak amplitude) were analyzed using SPSS or R software, and graphs were made in Excel. One-way ANOVAs were used to assess differences in b- and d-wave responses for each age and compound. Results were considered significant if the p-value was ≤0.05.

**FIGURE 1:**
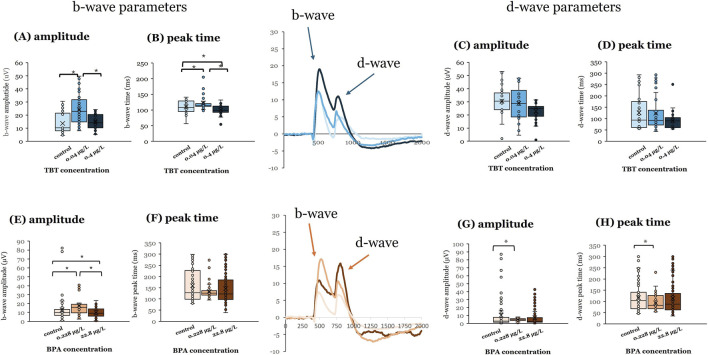
BPA or TBT exposure at 3 dpf enhances ON-bipolar responses. Electroretinograms (ERGs) were recorded from adult eyecups to assess retinal function. Zebrafish larvae were exposed to bisphenol A (BPA) at either 0.228 μg/L or 22.8 μg/L, (control = 0.0003% DMSO) or tributyltin (TBT) at 0.04 μg/L or 0.4 μg/L (control = 0.1% ethanol) for 24 h when they were 3 days postfertilization (dpf). Larvae were then placed into system water and allowed to grow to adulthood (≥3–4 months pf) when ERGs were recorded. ERGs were elicited in response to a white light stimulus at 7 different brightness levels. Responses shown are from the brightest stimulus level (ND3.0) as that is the largest response. Representative mean ERGs at the brightest stimulus level are shown in the center of the figure. ERG b-wave amplitude and peak time and d-wave amplitude and peak time were quantified. **(A)** b-wave amplitude, **(B)** b-wave peak time, **(C)** d-wave amplitude, and **(D)** d-wave peak time measured in adults developmentally exposed to TBT (blue bars). **(E)** b-wave amplitude, **(F)** b-wave peak time, **(G)** d-wave amplitude, and **(H)** d-wave peak time measured in adults developmentally exposed to BPA (orange bars). Significant differences (asterisks) were determined for each parameter using a one-way ANOVA evaluated at α = 0.05. (Parts of this figure are taken from Cohen et al. (2025) and Jensen et al. (2025).

For both compounds, exposure to the lower concentration (0.228 μg/L BPA; 0.04 μg/L TBT) at 3 dpf had greater effects than the higher concentrations (Figure 1), revealing non-monotonic effects. Exposure to 0.04 μg/L TBT at 3 dpf increased b-wave response amplitude (Figure 1A, p < 0.001) and b-wave peak time (Figure 1B, p = 0.019) compared to vehicle controls and 0.4 μg/L TBT exposed tissue. However, TBT exposure at 3 dpf did not alter OFF-bipolar d-wave responses (Figures 1C,D; N’s for the different groups: vehicle = 6 | 0.04 μg/L TBT = 6 | 0.4 μg/L TBT = 6). Exposure to 0.228 μg/L BPA at this age increased ON-bipolar cell b-wave (Figure 1E; p < 0.001) and OFF-bipolar cell d-wave (Figure 1G; p = 0.003) response amplitudes; d-wave response time was also faster than vehicle controls (Figure 1H; p = 0.005; vehicle = 10 | 0.228 μg/L BPA = 6 | 22.8 μg/L BPA = 18). Exposure to 22.8 μg/L BPA at 3 dpf decreased b-wave amplitude (p < 0.001).

**FIGURE 2:**
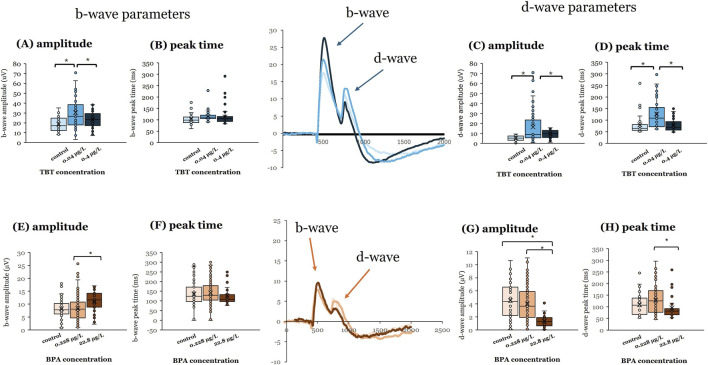
BPA or TBT exposure at 7 dpf affects OFF-bipolar cell responses. Electroretinograms (ERGs) were recorded from adult eyecups to assess retinal function. Zebrafish larvae were exposed to bisphenol A (BPA) at either 0.228 μg/L or 22.8 μg/L, (control = 0.0003% DMSO) or tributyltin (TBT) at 0.04 μg/L or 0.4 μg/L (control = 0.1% ethanol) for 24 h when they were 7 days postfertilization (dpf). Larvae were then placed into system water and allowed to grow to adulthood (≥3–4 months pf) when ERGs were recorded. ERGs were elicited in response to a white light stimulus at 7 different brightness levels. Responses shown are from the brightest stimulus level (ND3.0) as that is the largest response. Representative mean ERGs at the brightest stimulus level are shown in the center of the figure. ERG b-wave amplitude and peak time and d-wave amplitude and peak time were quantified. **(A)** b-wave amplitude, **(B)** b-wave peak time, **(C)** d-wave amplitude, and **(D)** d-wave peak time measured in adults developmentally exposed to TBT (blue bars). **(E)** b-wave amplitude, **(F)** b-wave peak time, **(G)** d-wave amplitude, and **(H)** d-wave peak time measured in adults developmentally exposed to BPA (orange bars). Significant differences (asterisks) were determined for each parameter using a one-way ANOVA evaluated at α = 0.05. (Parts of this figure are taken from Cohen et al. (2025) and Jensen et al. (2025).

Exposure to 0.04 μg/L TBT at 7 dpf also affected adult ERG responses (Figure 2). This TBT concentration increased b-wave (Figure 2A; p ≤ 0.001) and d-wave response amplitudes (Figure 2C; p ≤ 0.001) and delayed d-wave peak time (Figure 2D; p ≤ 0.001; vehicle = 4 | 0.04 μg/L TBT = 7 | 0.4 μg/L TBT = 5). For all three variables, responses measured after 0.04 μg/L TBT exposure were significantly different from both vehicle controls and the 0.4 μg/L treatment group. In contrast to TBT, it was the higher BPA concentration (22.8 μg/L) that significantly altered ERG responses in adult retinas when exposure occurred at 7 dpf. ERG b-wave amplitude was increased (Figure 2E; p < 0.001) compared to the 0.228 μg/L treatment group, whereas d-wave response amplitude was decreased (Figure 2E; p < 0.001) compared to both 0.228 μg/L BPA and vehicle controls. Exposure to 22.8 μg/L BPA at 7 dpf also quickened (reduced) the response time of the d-wave peak compared to the 0.228 μg/L BPA treatment group (Figure 2H; p = 0.02; vehicle = 6 | 0.228 μg/L BPA = 13 | 22.8 μg/L BPA = 5). Time to b-wave peak was not affected by either BPA (Figure 2F) or TBT exposure at 7 dpf (Figure 2B).

### 6.3 Developmental exposure to TBT or BPA caused age-dependent differences in retinal anatomy

**FIGURE 3:**
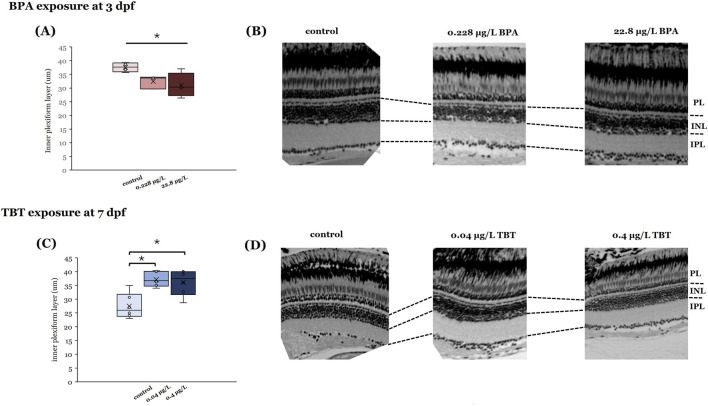
Effects of BPA and TBT on retinal anatomy are age dependent. Retinal sections taken from adults developmentally exposed to either BPA **(A,B)** or TBT **(C,D)** to determine if there are changes in retinal structure. Measurements of individual retinal layers (at the right) identified significant differences in the inner plexiform layer (IPL) only. Adults exposed to BPA when they were 3 days postfertilization (dpf) **(A,B)** showed a significant reduction in IPL thickness. In contrast, adults exposed to TBT when they were 7 dpf **(C,D)** showed a significant increase in IPL thickness. Measurements were made from 3 different retinas per treatment group per age. Significant differences (asterisks) were determined for each parameter using a one-way ANOVA evaluated at α = 0.05. (Parts of this figure are taken from Cohen et al. (2025) and Jensen et al. (2025).

To determine if the observed physiological differences reflected compound-induced changes in retinal anatomy, we measured the thicknesses of the different layers in H&E stained adult retinal sections (Figure 3). The only retinal layer found to be sensitive to compound exposure was the IPL, where axon terminals of ON- and OFF-bipolar cells are located. The anatomical change in retinal layer thickness for the IPL was different for the two compounds. BPA exposure at 3 dpf, but not 7 dpf, significantly decreased IPL thickness in adult retinas (Figure 3A; p = 0.042). In contrast, TBT exposure at 7 dpf significantly increased IPL thickness (Figure 3B; p = 0.002).

### 6.4 Developmental EDC exposure altered protein and mRNA expression of estrogen signaling components

We recently reported (Cohen et al., 2025) changes in estrogen signaling components in adult retinas following exposure to BPA at either 3 dpf or 7 dpf. Retinal homogenates collected from adults exposed to 0.228 μg/L BPA at 3 dpf showed a significant increase in mRNA expression of *aromatase (cyp19a1b)*, and a significant decrease in protein levels of ERβ, p-ERK and p-JNK (Cohen et al., 2025) indicating effects on genomic/nuclear signaling and the activation of either membrane bound ER or GPER (Prossnitz and Barton, 2014). In contrast, retinal homogenates from adult fish exposed when they were 7 dpf did not show differences in *aromatase*, *esr1*, or *esr2a* expression though ERβ protein levels were increased. Exposure to 0.228 μg/L BPA at 7 dpf decreased p-ERK levels, while exposure to 22.8 μg/L BPA increased p-JNK levels. The two BPA concentrations also differentially effected p-AKT levels, with increased p-AKT protein observed in retinas treated with 22.8 μg/L BPA but decreased protein levels observed in the 0.288 μg/L treatment group (Cohen et al., 2025). BPA-induced changes in AKT levels are important to note, as this pathway is associated with GPER activation and neuroprotection in mouse retina (Jiang et al., 2019).

We have not completed a similar molecular analysis of retinal homogenates from adults developmentally exposed to TBT, and no similar analysis is reported in the literature. However, we can anticipate TBT-induced effects on estrogen signaling components, as a transcriptomic analysis of whole zebrafish embryos exposed from 2 to 5 dpf revealed TBT exposure altered genes involved in development and immune and inflammatory responses (Martinez et al., 2020). Further, given that TBT is an aromatase inhibitor (Matthiessen and Gibbs, 1998; McAllister and Kime, 2003; McGinnis and Crivello, 2011) and that aromatase and ER are present in zebrafish retina (Menuet et al., 2002; Sawyer et al., 2006; Cohen et al., 2025), it is likely that estrogen levels/signaling will be reduced by early life TBT exposure, as reported in juvenile Atlantic salmon (Lyssimachou et al., 2006).

## 7 ERGs reveal persistent effects of BPA and TBT that were concentration and age dependent

**FIGURE 4:**
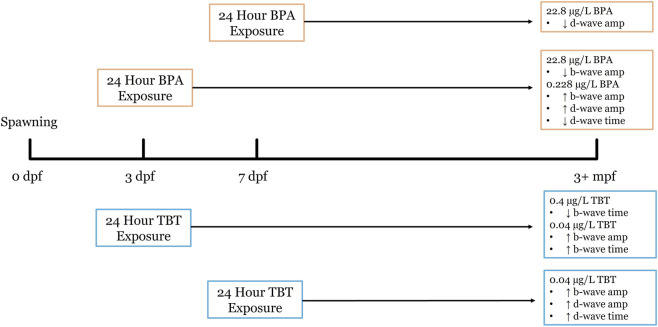
Developmental exposure timeline for BPA and TBT and resulting long-term retinal functional outcomes in zebrafish. This timeline illustrates the experimental design used to assess the long-term effects of early life exposure of BPA and TBT at our two exposure timepoints. Embryos were maintained in an incubator from 0 days post fertilization (dpf) to 10 dpf, before being transferred in the aquatic care facility, where they remained until 3 months post fertilization (mpf). Both BPA and TBT had 24 h exposures beginning at either the 3 dpf or the 7 dpf. Electroretinography (ERG) was performed at 3+ mpf to quantify long-term change in bipolar cells. High concentrations of BPA (0.1 μM) at 7 dpf result in an increase in b-wave amplitude and a decrease in d-wave time. While low concentration (0.228 ug/L) at 3 dpf BPA exposure increase b-wave amplitude and d-wave amplitude, with a decrease in d-wave time. High concentration of TBT (0.4 μg/L) at 7 dpf showed an increase in b-wave amplitude, d-wave amplitude, and d-wave time. As opposed to high concentration at 3 dpf which showed a decrease in b-wave amplitude.

Our data suggests similarities in functional outcomes to early life EDC exposure in zebrafish retina with respect to age of exposure and effective concentration for both BPA and TBT. ERG recordings from adult retinas following developmental exposure to BPA or TBT, identified age-dependent effects (Figure 4). In general, the ON-bipolar cell b-wave was sensitive to both compounds, regardless of exposure age. The OFF-bipolar d-wave, in contrast, appeared more sensitive to compound exposure at the later exposure age. Exposure to either 0.228 μg/L BPA or 0.04 μg/L TBT at 3 dpf enhanced adult b-wave amplitudes. This TBT concentration also delayed b-wave peak time but had no effect on d-wave responses. BPA, on the other hand, increased and delayed OFF-bipolar d-wave responses. These effects of TBT and BPA exposure at 3 dpf are evident in the mean traces shown in Figure 1. Thus, only ON-bipolar cells appear sensitive to TBT exposure at 3 dpf; while both ON and OFF-bipolar cells are sensitive to BPA. Exposure to either compound at 7 dpf affected both the b-wave and d-wave components of the adult ERG, with all OFF-bipolar response components showing significant differences. Adult retinas exposed to 0.04 μg/L (the lower concentration) TBT showed increased and delayed d-wave responses, as did ERG b-waves. In contrast, significant differences in adults previously exposed to BPA were only observed in the high (22.8 μg/L) treatment group which decreased d-wave amplitude compared to vehicle controls but increased b-wave amplitude. These age-dependent effects were also evident in the anatomical data. BPA exposure at 3 dpf also reduced the thickness of the retinal IPL; while TBT exposure at 7 dpf increased IPL thickness.

We also identified concentration-dependent effects. Developmental exposure to 0.04 μg/L TBT appears more deleterious than 0.4 μg/L as all ERG components were enhanced by the lower concentration, regardless of exposure age. Similarly, exposure to the lower 0.228 μg/L BPA concentration at 3 dpf consistently enhanced all ERG responses. These results describe nonmonotonic responses, as exposure to the lower dose caused more effects (Canesi and Fabbri, 2015). We did observe changes in ERG responses in adults exposed to the 22.8 μg/L BPA treatment, however, with this dose enhancing b-wave amplitudes but reducing d-wave responses when exposure occurred at the later age. Nonmonotonic effects of EDC’s have been reported (Vandenberg, 2014) including in retina (Kang et al., 2024). The suggested mechanisms underlying these responses include different affinities for/differential activation of receptors (Villar-Pazos et al., 2017; Vandenberg, 2022) and differences in pathway activation between central EDC effects, which would trigger regulatory negative feedback, and peripheral EDC effects, which would be stimulatory (Shi et al., 2025). G-protein coupled receptors can be desensitized, where continued exposure to an EDC inactivates the receptor, causing larger concentrations to be less effective (Vandenberg, 2022). In contrast, nuclear receptors can be downregulated as EDC concentration increases, decreasing the effects of higher doses (Vandenberg, 2022). These mechanisms all describe the impact of the EDC binding to a receptor, which is the case for BPA and ER. In fact, nonmonotonic responses of BPA are well reported, occurring in ∼20% of reports (Vandenberg, 2014). Given that BPA is known to have differential affinities for ERα/β (Ben-Jonathan and Steinmetz, 1998; Vandenberg et al., 2009) and that BPA can bind GPER, we suggest that differential binding to these receptors and/or receptor desensitization may underlie the BPA-induced nonmonotonic effects observed here. Determining the mechanisms of TBT’s nonmonotonic effects is more difficult, as TBT does not bind ER. Here, the observed responses may reflect competition between TBT and the natural substrate for the enzyme active site. Further, the ability of both BPA and TBT to bind thyroid receptors, and influence other cellular mechanism, must also be considered, as nonmonotonic responses can also occur when a single outcome (such as retinal function) is influenced by more than one pathway (Vandenberg, 2022).

**FIGURE 5:**
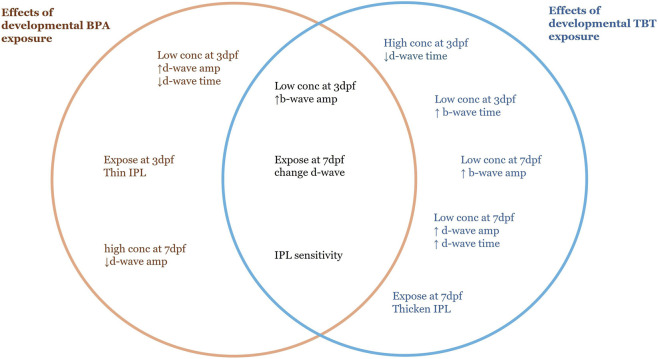
Summary diagram comparing the effects of developmental BPA and TBT exposure. Items on the left (orange text) were observed only in adult retinas developmentally exposed to BPA. Items on the right (blue text) were observed only in adult retinas developmentally exposed to TBT. Items in the center (black text) are common to both. Differences were in comparison to the relative vehicle control. For BPA: low conc = 0.228 μg/L; high conc = 22.8 μg/L; control = 0.0003% DMSO. For TBT: low conc = 0.04 μg/L; high conc = 0.4 μg/L; control = 0.1% ethanol. ↑, increase in amplitude or time; ↓, decrease in amplitude or time; Dpf, days postfertilization; IPL, inner plexiform layer.

The increase in b-wave amplitude in response to the lower concentrations of both BPA and TBT reveals similar, not opposite effects on retina function (Figure 5). While unexpected, we identified three studies with similar results. Antagonistic effects were not observed in neonatal (PN 1–16) female rats after co-exposure to a BPA/TBT mixture (Yang et al., 2019). Chronic (2–5 dpf) exposure to BPA and TBT in zebrafish revealed only concentration dependent effects, with higher doses of TBT needed to produce the same effects as low BPA (Martinez et al., 2019). Finally, a metabolomic analysis of zebrafish larvae exposed to either compound from 48 to 120 hpf revealed some overlap with regard to which metabolites/metabolic pathways were altered (Ortiz-Villanueva et al., 2018). These reports, together with our data, suggest that BPA and TBT may be differentially targeting the same and/or different pathways. These pathways, which include estrogen disruption, are described below.

## 8 Possible mechanisms of long-term functional effects resulting from acute developmental EDC exposure

### 8.1 Developmental exposure to BPA or TBT alters estrogen signaling pathways in retina causing latent physiological changes

Exposure to BPA and TBT, if only transiently, likely had a direct effect on retinal estrogen signaling. All estrogen receptor types are present in vertebrate retinas (Cascio et al., 2015) and aromatase is found in rat INL and OPL (Valero-Ochando et al., 2024) where bipolar cells and their dendrites are located. Importantly, estrogen signaling components are present in zebrafish retina at our exposure ages, including nuclear ER (Bardet et al., 2002; Lassiter et al., 2002; Tingaud-Sequeira et al., 2004), aromatase (Mouriec et al., 2009b; Cohen et al., 2025) and GPER (Shi et al., 2013). Developmental BPA exposure reduces eye size (Crowley-Perry et al., 2021; Volz et al., 2024) and affects brain development in zebrafish (Nakamura et al., 2006; Vandenberg et al., 2009; Zhou et al., 2009; Kim et al., 2011; Tse et al., 2013). Similarly, developmental TBT exposure delays eye development (Hano et al., 2007) and has retina-specific effects (Fent and Meier, 1992; Wang and Huang, 1999; Wester et al., 2004; Dong et al., 2006).

Our molecular analysis identified *aromatase (cyp19a1b)* and ER (*esr1*, *esr2a*) mRNA and/or protein (ERβ) in adult zebrafish retinal homogenates and found that developmental BPA exposure altered expression of some of these estrogen responsive genes (Cohen et al., 2025). We also observed differences in MAPK and AKT pathway activation (phosphorylation) suggesting BPA was also binding to GPER. In mammalian retina activation of GPER and/or ERβ is neuroprotective to retinal ganglion cells (Jiang et al., 2019; Rodriguez-Ramirez et al., 2025). BPA exposure (0.228 μg/L) at 3 dpf decreased ERβ, but increased p-ERK and p-JNK levels; while exposure to the same concentration at 7 dpf increased ERβ protein and decreased p-ERK, suggesting age-dependent differences ER/GPER activation by 0.228 μg/L BPA. BPA concentration-dependent effects were also observed for AKT pathway activation, with an increase in p-AKT (associated with pathway activation) found in retinas treated with 22.8 μg/L at 7 dpf and decreased levels in the 7 dpf 0.228 μg/L group (Cohen et al., 2025). GPER activation associated with PI3K/AKT pathway activation is neuroprotective (Jiang et al., 2019), suggesting a possible effect of BPA exposure. Together, this data suggests that a brief early life BPA exposure can cause latent effects on estrogen pathways in retina, with BPA concentration determining which specific ER/GPER is/are activated.

With regard to TBT, the presence of aromatase in zebrafish (Cohen et al., 2025) and mammalian (Cascio et al., 2015) retinas suggests a direct effect on estrogen synthesis. In his review of estrogenic signaling in mammalian retina, Cascio et al. (2015) indicates the retinal INL is the layer where most local E_2_ is synthesized. Goldfish retina contains aromatase-positive bipolar, horizontal, and amacrine cells in the INL (Gelinas and Callard, 1993; Callard et al., 1995), suggesting E_2_ synthesis also occurs in the INL in fish retina. Importantly, the INL is where bipolar cells are located. The changes in ERG b- and d-waves, which reflect bipolar cell responses, in reponse to TBT exposure suggest the INL may be targeted by compound exposure. Inhibition of aromatase changed the thickness of the IPL and INL in zebrafish and rats (Salyer et al., 2001; Orger and Baier, 2005) in support of this hypothesis. The IPL is where ERβ is located in mammals (Cascio et al., 2015) and our anatomical data indicates that the IPL is sensitive to TBT (and BPA) exposure, suggesting that TBT may selectively target the inner retina.

### 8.2 Long-term retina-specific effects of developmental BPA/TBT exposure are estrogen independent

While BPA and TBT are classified as EDCs, both compounds have a variety of effects. TBT exposure induces oxidative stress (Zhang et al., 2017) and increases levels of glucose and creatine kinase in the blood (Li and Li, 2021). TBT exposure increased permeability of the blood brain barrier, lipid peroxidation, glial activation and autophagy in rats (Mitra et al., 2013; Mitra et al., 2015). TBT exposure also causes apoptosis in isolated mammalian cells (Correia et al., 2025) and disrupts thyroid signaling in mice (Sharan et al., 2014), rats (Rodrigues-Pereira et al., 2022) and zebrafish (Li and Li, 2021). BPA can binding to thyroid receptors (Almeida et al., 2018; dos Santos et al., 2022) and to thyroid hormone transport proteins such as transthyretin, displacing thyroxine (T4) (Lim et al., 2002; Fujimoto et al., 2004; Brent, 2023). BPA working through thyroid hormone receptor β (*thrb*), in particular, is required for proper development of cone photoreceptors in zebrafish (Suzuki et al., 2013; Qiu et al., 2025) and mice (Roberts et al., 2006).

Other reports indicate BPA and TBT exposure alter levels of the neurotransmitters glutamate, dopamine, and GABA (Aoshima et al., 2001; Tsunoda et al., 2006; Choi et al., 2007; Almeida et al., 2018; Liu et al., 2020; Tu et al., 2020; Hyun and Ka, 2024). BPA and TBT impact these neurotransmitter systems by either blocking compound synthesis or binding to receptors. In *Xenopus* oocytes (Aoshima et al., 2001) and dissociated rat CA3 neurons (Choi et al., 2007), BPA had concentration dependent effects on GABA_A_ receptor mediated responses. In rodent brain, BPA decreased TH-immunoreactivity (Ishido et al., 2007). GABA and dopamine synthesis decreased, and glutamate synthesis increased, in zebrafish larvae exposed to BPA from 5 to 8 dpf (Kim et al., 2020). Similarly, adult male zebrafish exposed to TBT had decreased expression of genes involved in dopamine, GABA and serotonin synthesis (Tu et al., 2020). Developmental TBT exposure blocked ligand (glutamate) binding to NMDA receptors in rodent brain membrane preparations (Konno et al., 2005).

As in the rest of the brain, retinal signaling depends on the release of neurotransmitters. Photoreceptor synapses onto bipolar and horizontal cells are glutamatergic, with glutamate release decreasing in response to light stimulation–the basis for ON and OFF bipolar cell responses (i.e., ERG b- and d-waves, respectively). Zebrafish retinal bipolar cells also express GABA and dopamine receptors (Connaughton, 2011). In zebrafish retina, GABA is localized to horizontal cells in outer retina and amacrine cells in inner retina (Connaughton et al., 1999; Yazulla and Studholme, 2001; Marc and Cameron, 2002). Dopamine is present in retinal amacrine and interplexiform cells (Connaughton et al., 1999; Yazulla and Studholme, 2001; Marc and Cameron, 2002). Amacrine and interplexiform processes are found in the IPL, the retinal layer found to be sensitive to BPA and TBT exposure. Thus, BPA/TBT exposure could be impacting bipolar cell responses by changing modulatory (GABA, dopamine) inputs or altering photoreceptor glutamate release.

Dopamine is synthesized in a 3-step sequence from the amino acid phenylalanine, with tyrosine hydroxylase (TH) the rate limiting enzyme in the pathway (Daubner et al., 2011). GABA is synthesized from glutamate via the enzyme glutamic acid decarboxylase (GAD) (Zhang et al., 2024). We observed changes in the protein levels of both enzymes in adult retinal homogenates that were exposed to 0.228 μg/L BPA exposure at 3 dpf. TH protein levels were increased, while GAD levels were decreased (Cohen et al., 2025). These enzymes are the rate limiting enzymes in the synthesis of dopamine and GABA, respectively, suggesting a direct BPA-induced effect on neurotransmitter synthesis in exposed retinas. Exposure to the higher BPA concentration (22.8 μg/L) at 3 dpf increased GAD levels in the same homogenates, identifying concentration dependent effects.

In amphibian retina, blocking GABA_A_ receptors increased ERG b- and d-wave amplitudes (Hirasawa et al., 2021). We observed a thinner IPL and a decrease in GAD protein levels in adult retinal homogenates developmentally exposed to 0.228 μg/L BPA at 3 dpf (Cohen et al., 2025), suggesting a BPA-induced decrease in GABA synthesis, which may contribute to the enhanced b- and d-wave responses that were observed. Adult retinas treated with the higher concentration of BPA (22.8 μg/L) at 3 dpf displayed reduced b-wave amplitude but increased GAD protein levels, consistent with an increase in GABAergic inhibition. Both concentrations of BPA increased TH protein levels in retinal homogenates exposed at 3 dpf (Cohen et al., 2025), suggesting a BPA-induced increase in dopamine levels. Elevated dopamine levels reduce gap junctional coupling of (McMahon, 1994) and block GABA release from retinal horizontal cells (Yazulla and Kleinschmidt, 1982; Calaza et al., 2006), contributing to the observed ERG responses. TBT exposure similarly influences both dopamine and GABA. We observed an increase in IPL thickness due to TBT exposure at 7 dpf. This could suggest an increase in GABA and/or dopaminergic inner neurons and their synaptic connections or an increase in bipolar cell synaptic contacts. TBT is reported to increase dopamine levels in zebrafish (Liu et al., 2020) but decrease GABA levels (Tu et al., 2020). The increase in ERG response amplitudes we observed in retinas exposed to the 0.04 μg/L TBT treatment suggest a decrease in inhibition, consistent with these reports.

Alternatively, BPA/TBT could be targeting photoreceptor responses, causing a downstream effect on the postsynaptic bipolar cells which would alter b- and d-wave responses. Examining ERG a-wave response amplitudes and timing suggest this possibility (Cohen et al., 2025; Jensen et al., 2025). For example, exposure to 0.228 μg/L BPA at 3 dpf increased both b-wave and d-wave amplitudes. Photoreceptor a-wave amplitude was also increased in this BPA treatment group, suggesting a downstream effect. Similarly, exposure to 22.8 μg/L BPA at 7 dpf quickened both a-wave and d-wave implicit times. Exposure to the higher TBT concentration (0.4 μg/L) at 3 dpf similarly quickened b-wave and a-wave peak times. The lower TBT concentration (0.04 μg/L) at 7 dpf increased and delayed adult a-wave and d-wave responses. These similarities suggest that, for some of the outcomes measured, the change in bipolar cell responses may be indirect and downstream of a direct impact on photoreceptors. BPA exposure in zebrafish larvae altered red (L) and UV cone morphology and disrupted the retinal cone mosaic (Qiu et al., 2025), suggesting a direct effect of BPA on photoreceptors. Photoreceptor sensitivity to TBT is highly likely, given the localization of aromatase to the ONL in some vertebrate retinas (Valero-Ochando et al., 2024) and the identification of *aromatase (cyp19a1b)* in zebrafish retina (Cohen et al., 2025).ERG.

Taken together, the changes in ERG b- and d-wave responses in adult retinas from fish developmentally exposed to BPA or TBT could be due to EDC-induced changes in neurotransmitter release/levels within the retina.

### 8.3 BPA and TBT-induced changes in retinal function are due to a direct effect on retinal bipolar cells

Finally, both BPA and TBT are known to directly alter neuronal activity, suggesting that the ERG differences observed may reflect a direct effect of these EDCs on retinal bipolar cells. TBT application can increase calcium levels in isolated DRG neurons (Fross et al., 2021) and reduce Na^+^/K^+^ pump activity in brain (Zhang et al., 2008; Li et al., 2015). BPA inhibited L-, N-, and T-type Ca^+2^ channels in isolated GH3 cells and reduced L-type Ca^+2^ channel amplitude in cardiac myocytes (Deutschmann et al., 2013) and rat aortic smooth muscle (Feiteiro et al., 2018). These actions of BPA are due to the compound binding to and stabilizing calcium channels in the resting state (Deutschmann et al., 2013). BPA application increases the amplitude of BK potassium channels in AD 293 cells by binding to both intracellular and extracellular sites on the channel (Rottgen et al., 2014), and inhibits TTX-sensitive and TTX-insensitive Na^+^ channels in DRG neurons via PKA and PKC dependent pathways (Wang et al., 2011). Zebrafish bipolar cells express depolarization elicited calcium (T-type or L-type) and potassium (sustained I_K_ or transient I_A_) channels (Connaughton and Maguire, 1998). Patch clamp analysis of adult zebrafish bipolar cells revealed BPA-induced effects (Cohen et al., 2025). Developmental BPA exposure reduced L-type Ca^+2^ current amplitude regardless of exposure age; whereas outward rectifying K^+^ currents (I_K_) were reduced when developmental BPA exposure occurred at 3 dpf but increased when exposure occurred at 7 dpf. These differences in voltage gated currents measured in tissue collected from adults at the later age were mostly observed in response to 22.8 μg/L BPA exposure, suggesting concentration-dependent effects, as reported in other preparations (Wang et al., 2011; Deutschmann et al., 2013; Feiteiro et al., 2018). In isolated pancreatic β-cells, concentration dependent effects of BPA on R-type Ca^+2^ channels were reported, with exposure to a low (1 nM) dose of BPA decreasing current amplitude (Villar-Pazos et al., 2017). In this preparation, BPA did not directly interact with the Ca^+2^ channel, but induced effects through differential activation of ERβ and ERα (Villar-Pazos et al., 2017).

Changes in voltage gated currents across a population of retinal cells would affect ERG responses. Increases in b-wave amplitude, for example, could reflect increased internal calcium levels, as reported to occur after TBT exposure (Fross et al., 2021). A reduced current through voltage-gated calcium channels coupled with a reduced outward rectifying K^+^ current would lower overall bipolar cell responses. Alternatively, a reduced calcium current and enhanced outward K^+^ current would reduce and delay neuronal responses. These latter options were observed in BPA treated adult retinas.

## 9 Translational implications and future directions

Endocrine disrupting compounds are a public health concern worldwide because of persistent exposure, diverse point sources, and the presence of significant low dose effects (Niehs, 2025). Two well-known EDCs, BPA and TBT, are of primary concern and were the focus of this review. BPA is ubiquitous as it is still continually used for industrial and household products. The effect of BPA on animals is well-reported and recent reports show effects of early life BPA exposure in humans (Lin et al., 2023; Hyun and Ka, 2024; Triswindyaningrum et al., 2025). In contrast, while TBT use has been banned for almost 20 years, the slow degradation of this chemical coupled with recent increases in environmental concentrations and measurable levels in humans (Correia et al., 2025) keep TBT a chemical of concern.

Our goal was to review estrogen signaling in retina and identify how BPA and TBT exposure alter these pathways. These compounds have opposite effects on estrogen signaling pathways. Interestingly, it does not appear that exposure to these compounds cause opposite outcomes. Developmental exposure to either compound showed similar effects on adult retinal ERG responses, with enhancement of ON-bipolar cell b-wave responses observed for the lower concentrations used. While we did observe a few contrasting outcomes, they depended on age of exposure (i.e., IPL thickness) and/or concentration (i.e., d-wave responses). It is important to note that while environmentally relevant levels of both compounds were used, these levels were several orders of magnitude different from each other. Nonetheless, both compounds displayed developmental effects. While bath application of the compounds likely resulted in systemic differences, our focus on the retina identifies specific effects on neuronal signaling in this tissue. Current experiments are continuing to elucidate the long-term effects of these compounds by examining changes in gene expression, individual neuronal responses, and larval differences.

Overall, our findings suggest that the retina is extremely sensitive to EDCs, and that adverse outcomes due to BPA and/or TBT exposure should include analysis of long-term developmental effects focused on the visual system.
